# Supplementary material for: AI-Enabled Diagnostic Prediction within Electronic Health Records to Enhance Biosurveillance and Early Outbreak Detection
Source: Res Sq. 2025 Jun 12:rs.3.rs-6606632. Preprint. [Version 1] doi: 10.21203/rs.3.rs-6606632/v1 (PMC12204487; doi:10.21203/rs.3.rs-6606632/v1)
Supplement: 1 [file NIHPPrs6606632v1-supplement-1.pdf]

Supplementary Files

This is a list of supplementary files associated with this preprint. Click to download.

- SupplementaryInformation.docx
- Tables.docx
